# Reference levels of mean and peak anaerobic power for male and female long-track speed skaters

**DOI:** 10.5114/biolsport.2025.144407

**Published:** 2024-10-25

**Authors:** Dariusz Sitkowski, Michał Starczewski, Andrzej Klusiewicz

**Affiliations:** 1 Department of Physiology, Institute of Sport – National Research Institute, Warsaw, Poland; 2 Faculty of Rehabilitation, Józef Piłsudski University of Physical Education in Warsaw, Warsaw, Poland; 3 Faculty of Physical Education and Health, Biała Podlaska, Józef Piłsudski University of Physical Education in Warsaw, Warsaw, Poland

**Keywords:** Anaerobic capacity, Wingate test, Sprinting ability, Laboratory testing, High-level athletes, Age and gender differences

## Abstract

The ability to generate and maintain high power is a prerequisite for success in many sports, including long-track speed skating. A common method of determining this ability is the Wingate test, but surprisingly, normative data necessary to objectively assess the performance of speed skaters is lacking. Therefore, the present study aimed to develop reference levels for peak power (PP) and mean power (MP) on a Wingatetype test for speed skaters using a Monark 874E ergometer with a load equivalent to 7.5% of body mass. Over 15 years, 635 test results were collected from 120 junior and senior athletes (all national representatives); 380 from men and 255 from women. We found that in men and women, personal best in relative PP and MP (W/kg body mass) correlated significantly (all p < 0.001) with altitude-adjusted times over distances of 500 (r = -0.75 to -0.82) and 1000 m (r = -0.67 to -0.71), thus confirming the validity of this test for assessing the on-ice sprinting ability of speed skaters. Moreover, the two-way ANOVA models revealed significant effects of gender (p < 0.001) and age (p < 0.001) on relative MP and PP (W/kg) values, but the interaction effect was not significant (p > 0.05). The 7-stage classification system constructed on the basis of the obtained results enables the assessment of relative MP and PP in Wingate tests, taking into account the age category and gender of the athletes. This system can be used by sports researchers and coaches to assess the sprinting ability of speed skaters from novice to world-class levels.

## INTRODUCTION

Long-track speed skating is one of the most popular sports in the world and a stable feature on the Olympic Winter Games program since its inception in 1924. To determine what factors influence the exercise capacity and athletic performance of speed skaters, numerous studies have been conducted in the fields of anthropology, biomechanics, psychology, and sports physiology [1–3]. Although it was found that the performance of skaters was unrelated to anthropometric characteristics and power output seemed independent of body dimensions, the thigh musculature of elite skaters was more developed compared to students, marathon runners, sprinters, figure skaters, and non-elite speed skaters, which may reflect the importance of the power generated by hip and knee extensor muscles in speed skating [2, 4]. Moreover, a longitudinal study found that from the junior to senior age category, more successful skaters showed a faster development of mean power output on a Wingate-type test than their less successful counterparts [3].

Anaerobic metabolic processes are particularly important in speed skating sprint events. It has been estimated that at distances of 500 and 1000 meters, anaerobic metabolism covers 82 and 67% of energy expenditure, respectively [5]. A comparable contribution of anaerobic metabolism was demonstrated in the Wingate test [6, 7], which, like skating, primarily activates the knee and hip extensor muscles. Therefore, it can be assumed that the Wingate test has strong predictive value in speed skating, especially at sprint distances, and may also be useful in determining exercise potential and training gains among, speed skating candidates. Although some research with a homogeneous group of elite junior speed skaters did not show the seasonal variation of results in Wingate-type 30-s test or differences from senior athletes [8] and no consistent correlation with all-round skating performance [9], others have reported a direct association between relative power in this test and performance over 500 and 1500 m [10–12]. Moreover, it was recently demonstrated that the relative values of mean and peak power (in the Wingate test) during the summer training period were good predictors of improvements in 1500-m speed-skating performance during the following competitive season [13].

Apart from a few studies on other sporting representatives or nonathletes [14–17], there is still a lack of normative power data for the Wingate test among male and female speed skaters. A reference dataset would allow a more objective evaluation of the sprinting abilities of speed skaters (e.g., talent identification, seasonal assessments) or training-induced changes over time, and could provide a useful adjunct for developing predictive models. Therefore, to fill this gap, we conducted a retrospective analysis of 30-s Wingate test results, which is systematically performed in our laboratory by top Polish speed skaters, collected over 15 years. The purpose of this study was to determine reference levels of mean and peak power values in Wingate-type test for male and female long-track speed skaters across different age categories.

## MATERIALS AND METHODS

### Participants

The study included 120 (72 male, and 48 female) long-track speed skaters (aged 13.2 to 36.9 years) who were members of the junior or senior national teams of Poland. Among them were medalists at the Olympic Games and World Championships (both in the junior and senior age categories).

### Ethics

This study was approved by a local Ethics Committee (KEBN-2494-DS) and performed in accordance with the Declaration of Helsinki and its later amendments. All participants, and parents or legal guardians in the case of those under 18 years of age, provided an informed written consent before taking part in the study.

### Study design

The study design involved a retrospective analysis of Wingate power data from athletes who participated in a training monitoring program run by the Polish Speed Skating Association (PZŁS) and in a national Talent Identification program. Power output was determined from a standardized Wingate-type 30-s supramaximal exercise test, each performed on the same cycle ergometer under laboratory conditions. Data were collected during laboratory testing of athletes at the beginning (May) or end (September) of the general preparation phase of an annual training cycle (dry-land training periods) from 2007–2021.

### Methodology

The athletes spent the night before testing at a dormitory located in the laboratory building. The next day, after waking up and urinating, a body mass (BM) check was conducted with the minimum possible clothing. Athlete BM mass was measured with an accuracy of 50 g using an electronic scale (Dolphin, CAS Corp., South Korea). Next, no earlier than 90 min after a standardized breakfast, they underwent a medical examination, which included an inspection of medical certificates confirming their ability to practice competitive sports, a medical history, as well as electrocardiography, auscultation of the heart and lungs, and assessment of blood pressure. After ruling out health contraindications to strenuous exercise, the Wingate-type test was performed (always in morning sessions) on a mechanically-braked cycle ergometer (Monark 874E, Monark Exercise AB, Vansbro, Sweden) with pedals and saddle replaced from a road bike. The 30-s Wingate-type test was preceded by a 5-minute warm-up (on a replicate ergometer) with an individually selected load of 1.5–2.0 kg for men or 1.0–1.5 kg for women. During the warm-up, the pedalling rate was self-selected by the athletes, but they were instructed to maintain between 60–80 rpm, except a two 3–4-s bursts (executed after about 2.5 and 3.5 min) at a maximal pedalling rate. If the athletes did not feel warmed up enough, they could do an extra sprint and extend the warm-up by one minute. After the warm-up, the subject rested for about 4–5 min. At that time, the ergometer’s seat height was adjusted so that when pedalling the athlete’s knee angle (at extension) was about 25 degrees. The handlebar was also adjusted according to individual preferences. At the same time, a weight equivalent to 7.5% of the athlete’s BM (with a resolution of 100 g) was placed in the ergometer basket. Both male and female athletes were tested at the same relative load. Once declaring themselves ready for the test, the athletes sat on the ergometer and were helped to tighten the straps binding their feet to the pedal toe clips. They were then instructed to maintain a stationary position for a short period (with the crank arms at an angle about 45 degrees to the ground, left leg up, about 2 cm before the impulse sensor) and to prepare themselves mentally for maximal effort without lifting their hips off the seat. After the athletes signalled that they were ready to begin, a 3-s countdown was given followed by a command to “*Go*”. The first signal from the reed switch started the time measurement. Vigorous verbal encouragement was given throughout the 30-s test, and timing feedback was provided at 10, 5, and 3 s before exercise completion.

Mean power (MP) and peak power (PP) were determined at a sampling frequency of 1000 Hz using a commercially available MCE measurement system (JBA, Zb. Staniak, Poland). Peak power output was taken as the highest power value from the fastest single revolution, while MP output was taken as the average power attained throughout the test period. The athletes did not receive any visual or verbal feedback on the power generated.

### Data processing

A total of 635 test results were collected from the 120 study participants; 380 from men and 255 from women. To examine relationships between Wingate performance and actual sporting performance at sprint distances, and thus confirm test validity for assessing the sprinting ability of skaters, the highest MP and PP values (expressed in W and W/kg) for each athlete were extracted and compared to personal best times over the 500 m and 1000 m distances, which were retrieved from the PZŁS database (https://domtel-sport.pl/statystyka/baza/). To reduce the impact of factors that most strongly influence performance in speed skating (i.e. open/closed rink and altitude) [18], only results from indoor rinks were included in this analysis (500 m distance N = 62 and N = 42, 1000 m distance N = 58 and 43, in men and women, respectively). The times were also adjusted to the altitude at which they were obtained. This adjustment was based on data describing the relationship between altitude and percentage differences in times over the 500 and 1000 m distances, taking Calgary as the reference altitude [19]. The linear regression equations determined from these data were as follows:

Time difference 500 m (%) = -0.0019 × altitude (m) + 2.0145

Time difference 1000 m (%) = -0.0033 × altitude (m) + 3.4585

The second part of the study examined the effect of age and gender on relative MP and PP values (W/kg). For this purpose, the data collected were grouped into age categories according to the rules of the International Skating Union, which distinguishes the following categories: junior C (13–14 years), junior B (15–16 years), junior A (17–18 years) and senior (19 years and older). While the results of one athlete may have appeared several times in a given age category, we chose to focus on the best result achieved in each category. Subsequently, 213 results were used for age-group binning: 123 (N = 12, 26, 41, 44 in consecutive age groups) in men, and 90 (N = 14, 25, 28, 23) in women. Finally, following other published work [16], seven reference levels of relative MP and PP (W/kg) were established for each age group of men and women, using the mean and standard deviation (SD) change of ± 0.5 as follows: very high (> mean + 1.5 SD), high (> mean + 1.0 SD), above average (> mean + 0.5 SD), average (mean ± 0.5 SD), below average (< mean – 0.5 SD), low (< mean – 1.0 SD), very low (< mean – 1.5 SD).

### Statistical Analysis

Pearson correlations and linear regression analyses, including slope and intercept comparisons, were used to examine relationships between Wingate PP or MP values and performance at the 500 and 1000 m distances in male and female speed skaters. A two-way analysis of variance (ANOVA) was applied to analyze the main effects of age group and gender, as well as their interaction (age group × gender). Where appropriate, within-group changes and between-group differences were tested using Duncan’s post hoc test. Assumptions of normality and homogeneity of variance were verified by the Shapiro-Wilk test or Levene’s test, respectively. An alpha level of p < 0.05 was considered statistically significant. Statistical analyses were performed using Statistica software (version 13.3, TIBCO Software Inc.), except for slopes and intercepts comparisons, where MedCalc^®^ Statistical Software version 22.021 (MedCalc Software Ltd., Ostend, Belgium) was used.

## RESULTS

**FIG. 1:**
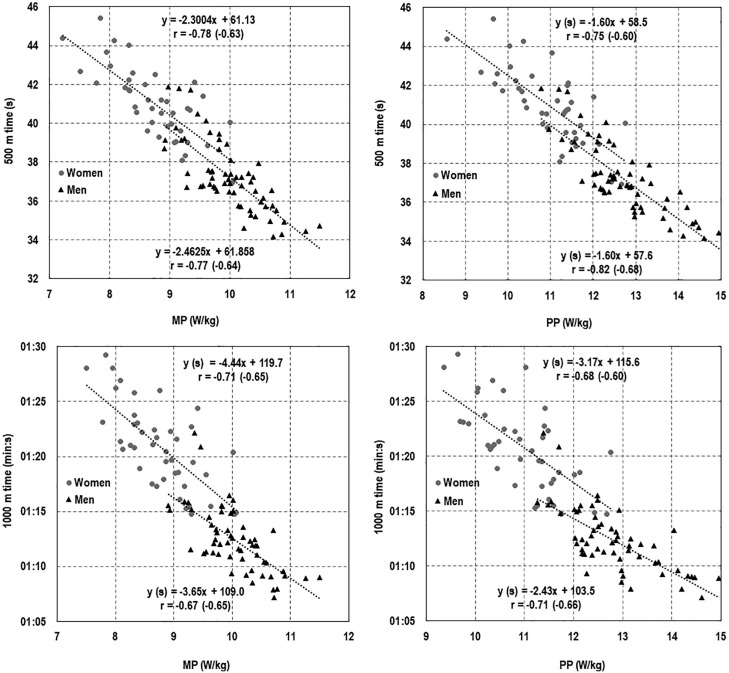
Relationships between altitude-adjusted times in the 500 and 1000 m races and relative values of MP or PP (W/kg) in the 30-s supramaximal exercise test on a cycle ergometer. Correlation coefficients with absolute values of MP and PP (W) are given in brackets. In the linear regression equations, *y* values were calculated in seconds.

Using regression analyses, strong to very strong negative correlations (all p < 0.001) emerged between relative MP or PP (in W/kg) and altitude-adjusted times over 500 and 1000 m distances in male and female speed skaters (fig. 1).

Altitude-adjusted times were also strongly associated with absolute MP and PP (W), but in all cases, the correlation coefficients (shown in brackets) were lower than that observed for relative values. There were no significant gender differences in the regression coefficients (p = from 0.220 to 0.986). However, the adjusted intercepts for men and women differed significantly over the 500 m (MP/kg – p = 0.013, PP/kg – p = 0.002) and 1000 m (MP/kg – p < 0.001, PP/kg – p < 0.001) distances.

**FIG. 2:**
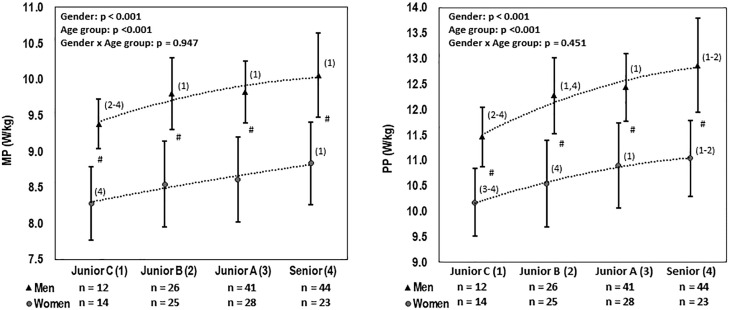
Relative values of MP and PP (W/kg) in the 30-s supramaximal exercise test on a cycle ergometer in male and female speed skaters representing different age categories (mean ± SD). Significant differences between groups 1–4 are indicated by numbers in brackets, and between gender by #.

The two-way ANOVA models revealed significant effects of gender and age on relative MP and PP (W/kg) values, but the interaction effect was not significant (fig. 2).

Reference levels of relative (W/kg) mean power (MP) and peak power (PP) in the 30-s supramaximal cycling test in male and female speed skaters across age categories are presented in Tables 1 and 2, respectively.

**TABLE 1:** Reference levels of relative values of mean power (MP) and peak power (PP) in the 30-s supramaximal exercise test on a cycle ergometer for male long-track speed skaters.

	PP (W/kg)				MP (W/kg)			

Reference levels	Junior C	Junior B	Junior A	Senior	Junior C	Junior B	Junior A	Senior
Very high	> 12.33	> 13.39	> 13.43	> 14.25	> 9.92	> 10.49	> 10.50	10.94
High	12.05–12.33	13.03–13.39	13.11–13.43	13.80–14.25	9.73–9.89	10.31–10.55	10.26–10.47	10.65–10.94
Above average	11.76–12.04	12.65–13.02	12.77–13.10	13.34–13.79	9.56–9.72	10.06–10.30	10.05–10.25	10.36–10.64
Average	11.16–11.75	11.89–12.64	12.09–12.76	12.40–13.33	9.21–9.55	9.55–10.05	9.61–10.04	9.76–10.35
Below average	10.87–11.15	11.52–11.88	11.76–12.08	11.94–12.39	9.04–9.20	9.30–9.54	9.40–9.60	9.47–9.75
Low	10.58–10.86	11.15–11.51	11.43–11.75	11.48–11.93	8.87–9.03	9.05–9.29	9.18–9.39	9.18–9.46
Very low	< 10.58	< 11.15	< 11.43	< 11.48	< 8.87	< 9.05	< 9.18	< 9.18

**TABLE 2:** Reference levels of relative values of mean power (MP) and peak power (PP) in the 30-s supramaximal exercise test on a cycle ergometer for female long-track speed skaters.

	PP (W/kg)				MP (W/kg)			

Reference levels	Junior C	Junior B	Junior A	Senior	Junior C	Junior B	Junior A	Senior
Very high	> 11.18	> 11.81	> 12.14	> 12.16	> 9.05	> 9.43	> 9.49	> 9.69
High	10.85–11.18	11.40–11.81	11.74–12.14	11.80–12.16	8.80–9.05	9.15–9.43	9.21–9.49	9.42–9.69
Above average	10.52–10.84	10.97–11.39	11.32–11.73	11.42–11.79	8.54–8.79	8.85–9.14	8.91–9.20	9.13–9.41
Average	9.84–10.51	10.11–10.96	10.48–11.31	10.66–11.41	8.02–8.53	8.25–8.84	8.31–8.90	8.55–9.12
Below average	9.50–9.83	9.69–10.10	10.06–10.47	10.29–10.65	7.77–8.01	7.95–8.24	8.02–8.30	8.26–8.54
Low	9.17–9.49	9.26–9.68	9.65–10.05	9.92–10.28	7.51–7.76	7.65–7.94	7.72–8.01	7.97–8.25
Very low	< 9.17	< 9.26	< 9.65	< 9.92	< 7.51	< 7.65	< 7.72	< 7.97

## DISCUSSION

Our analysis showed that the altitude-adjusted 500 and 1000 m times achieved by men and women in actual competition were correlated, strongly or very strongly, with MP and PP achieved in a 30-s supramaximal exercise (cycle) test performed in the laboratory. These associations were particularly robust when power was expressed per kg of BM, thereby confirming the validity of this laboratory test for assessing the on-ice sprinting ability of speed skaters. In addition, significant variations were found in MP and PP (W/kg) output between age groups, as well as between men and women belonging to the same age category, highlighting the discriminative utility of the classification categories adopted.

The majority of published 30-s Wingate test results for long-track speed skaters have involved Dutch elite athletes [1, 13]. However, they were tested using an electrically-braked cycle ergometer (Lode Excalibur Sport, Groningen, The Netherlands), which gives different power values than the mechanically-braked Monark ergometer [20], as used herein. Therefore, it would be misleading to directly compare our power results to these athletes. Using the same 30-s assessment on a Monark ergometer, Canadian national team members produced higher relative PP and MP values than our male speed skaters [11]. At the same time, our female skaters had comparable and higher power values than a Canadian cohort consisting of a sprintbased and all-round group, respectively. However, the use of higher resistance (11.2 kp/kg for male sprinters, 10.7 kp/kg for male allrounders, and 9.2 kp/kg for female skaters) may have enabled the Canadians to achieve higher power values than our competitors [21, 22]. On the other hand, a comparison with other athletes (both genders) participating in sports requiring explosive power, who were also tested on a Monark ergometer with a resistance load of 7.5% of BM [14, 16], showed that our speed skaters presented higher relative MP and PP (W/kg). Still, these differences may be due, at least partially, to differences in starting position; stationary (favorable) vs. flying [16].

Since anaerobic processes are the predominant source of energy in both the Wingate test and the 500- and 1000-m races [5–7], it may be argued that power generated in such a laboratory test would transfer to field- or competition-based performance. This assertion is supported by our findings of strong to very strong relationships between these outcomes. Early work (1980s and early 1990s) also demonstrated a relationship between power in a 30-s cycle ergometer test and 500 m performance, expressed as power generated [12] or speed [11]. Surprisingly, we were unable to locate more recently published literature on this topic. In contrast, among junior skaters, there was no clear evidence of a direct relationship between skating and 30-s Wingate test performance [9]. Here a relationship emerged, but only in one of two observation periods, and only in women did MP exhibit a significant relationship with total all-round points. More consistent correlations with skating performance were found with other technical variables, such as trunk position and push-off direction.

Skating technique and aerobic and anaerobic energy production are considered the main internal factors determining speed skating performance [1, 19]. In our study, the anaerobic performance indices (i.e. relative MP and PP) alone explained 57% to 67% and 45% to 50% of the total variance in altitude-adjusted times over the 500 m and 1000 m distances, respectively. It is worth noting that the relationship between speed skating and Wingate-type test performance was based on individual best results, which may better reflect the adaptive potential of each athlete rather than expected transitory shifts within and across one or more competitive seasons. Moreover, ice quality can not only vary from rink to rink but can also change during a single test session [19], potentially affecting relationships between on-ice and cycle ergometer performance. Repeated measurements may also have reduced the effect of familiarization with the laboratory test used [23]. However, it should also be noted that relatively large differences in the sporting level of the study participants may have contributed to the magnitude of the correlation/determination coefficients observed.

Cross-sectional studies are used to determine normative values for a given population [24]. However, in the case of separated age groups, these values may depend not only on participant age but also on the participation of athletes with different sports levels and sports specializations, which can be particularly problematic when the number of participants per subgroup is quite low. However, our research used a mixed design, in which the results of some athletes appeared in different age groups, which could reduce the effects of the unequal distribution of individuals with different anaerobic potential and better describe age-related changes.

In the present study, the mean values of relative MP and PP (W/kg) increased with age. This is finding consistent with research on speed skaters, as well as ice hockey or soccer players, in cross-sectional and longitudinal settings [3, 14, 25, 26], but contrary to other work on elite junior speed skaters, who found similar relative MP values in elite senior speed skaters [8]. Also, the gender differences in power output (higher values in men) are consistent with reports on speed skaters and athletes from disciplines requiring high power output [3, 13, 16]. These findings are consistent with the model intercepts, whereby the same power corresponds to worse times in women than in men at distances of 500 and 1000 meters. Although anaerobic power and capacity are not the only determinants of performance in speed skating, differences in intercepts may suggest less efficient skating techniques in women than in men. Both the differences we found between age categories and between genders in MP and PP justified the reference levels we adopted, which take into account the age and gender of athletes.

Some authors have presented classification systems for MP and PP in the Wingate test based on percentile norms [14, 15]. However, like others [16], half of the SD was used in developing such a system in our study to distinguish between the reference levels. This 7-level classification system, with 3 levels on each side of the average level, allows sports researchers and coaches to accurately assess the anaerobic power and capacity of male and female speed skaters belonging to different age categories.

The relatively small sample size is a limitation of this study, especially when attempting to establish reference levels for a specific population. However, to increase the sample size, it would be necessary to include athletes outside the national teams, which could lead to an underestimation of power outputs for elite speed skaters. Some have also argued that the load (braking force) applied in the Wingate test underestimates actual power generated [21, 22], although as a standard laboratory test in sport, it allows for comparisons across laboratories and studies. It is worth mentioning that the load applied (7.5% of BM) shows consistency across research [14, 16, 26] in which reference levels were determined, even in athletes representing sports with high peak power requirements. As another potential source of bias, reference levels were defined for boys and girls as young as 13–14 years old, with relatively short experience in sports training, so it remains unclear as to whether they will specialize in sprint or long-distance competitions in the future.

## CONCLUSIONS

This study confirmed the potential of the 30-s Wingate-type test, implemented on a mechanically-braked cycle ergometer Monark 874E with a load equivalent to 7.5% of body mass, to predict or discriminate the sprinting ability of male and female speed skaters based on altitude-adjusted 500 m and 1000 m times. The proposed classification system, which considers gender and age categories, allows researchers and coaches to more objectively assess this trait among speed skaters from novice to world-class levels.
